# Utility of first trimester obstetric ultrasonography before 13 weeks of gestation: a retrospective study

**DOI:** 10.11604/pamj.2017.26.121.10336

**Published:** 2017-03-02

**Authors:** Felix Uduma Uduma, Anelkan Abaslattai, Dianabasi Udoete Eduwem, Morgan Ekanem, Philip Chinedu Okere

**Affiliations:** 1 Department of Radiology, Faculty of Clinical Sciences, College of Health Sciences, University of Uyo, Uyo, Nigeria; 2 Department of Obstetrics and Gynecology, Faculty of Clinical Sciences, College of Health Sciences, University of Uyo, Uyo, Nigeria; 3 Department of Community Medicine, Faculty of Clinical Sciences, College of Health Sciences, University of Uyo, Uyo, Nigeria; 4 Department of Radiation Medicine, College of Medicine, University of Nigeria, Enugu Campus, Enugu, Nigeria

**Keywords:** First trimester, ultrasonography, gestational sac, viability

## Abstract

**Introduction:**

First trimester pregnancy is defined as twelve weeks after the last menstrual period. Ultrasonography has revolutionized validation and management of first trimester pregnancies. The aim was to analyze ultrasonographic findings of first trimester pregnancies in University of Uyo teaching hospital (UUTH), Uyo, Nigeria.

**Methods:**

The departmental ultrasonographic records of pregnant women who were referred to Radiology department of UUTH, Uyo, Nigeria. For ultrasound scans were retrospectively reviewed. The period under consideration was from 8^th^ January 2013 to 8^th^ February, 2016. Demographic data and ultrasonographic parameters of first trimester pregnancies like gestational sacs were recorded. Data were statistically analyzed using SPSS Chicago 13. Exclusion criterion included incomplete data and acyesis despite positive βhCG test.

**Results:**

26.4% (n-645) of the 2438 pregnant women who underwent obstetric ultrasonography had first trimester ultrasonography during the studied period. The peak frequency was seen in the 20-29 age range with 52.2% (n-337) and followed by 30-39 age range with 41.7% (n-269). The commonest first trimester ultrasound findings was viable pregnancy with 42.5% (n-274), followed by incomplete miscarriage with 34.3% (n-221). The least finding was trophoblastic pregnancies 0.3% (n-2). The earliest age at which normal viable pregnancy was diagnosed in this study was 5weeks 5days from the last menstrual period. Only 2.19% (n-6) of the normal viable pregnancies were multiple pregnancies and they were all twins. The highest number of referrals to Radiology Department for first trimester ultrasonography was from accident and emergency unit with 34.42% (n-222) while antenatal clinic referral was only 16.12% (n-104).

**Conclusion:**

The commonest first trimester’s obstetric ultrasonographic findings in Uyo, Nigeria are viable pregnancies and are predominantly single gestation. The earliest age of ultasonographic pregnancy detection in Uyo is 5week 5days and peak maternal age is second and third decades.

## Introduction

Ultrasonography has consistently become a milestone in obstetric management of all trimesters of pregnancy. Its utility in pregnancy has increased dramatically over the past three decades [1, 2]. In fact, it confers easiness both to the attending Doctors and the expectant mothers at all facets of the pregnancy. This is borne out of its non-invasiveness, non-ionizing nature, real imaging potential, affordability, fetal amenability and bed sidedness in contrast to other radiological modalities. It has rapidly replaced all other techniques used to study normal human development especially in the first trimester [3].The advent of high-resolution trans-vaginal ultrasound (TVS) has corroborated trans-abdominal ultrasonography (TAS) and went further to revolutionize our understanding of the pathophysiology and management of pregnancy [3]. First trimester pregnancy is defined as twelve weeks after the last menstrual period [4]. It is known to be the important period of organogenesis but could be fraught with high complication rate [4, 5]. This calls for a lot of dedication and circumspection on ultrasonographic evaluation in this crucial period. First and foremost, first trimester ultrasonography must establish the existence of cyesis before any other meaningful assessment could continue. This assessment will then aim at visualizing viability, dating pregnancy, detecting multiple pregnancy, evaluating normal embryonic/foetal development, evaluating foetal gross anomaly, assessing nuchal translucency/ other markers, observing adnexal structures, observing uterine/ cervical lesions and detecting other special indications [4]. Therefore mastery of the spectrum of sonographic findings in the normal and abnormal first-trimester pregnancy equips the radiologist with the potentials to make accurate diagnoses and assists in appropriately guiding patient management [6]. The aims were to analyze the ultrasonographic findings of first trimester pregnancies in University of Uyo teaching hospital, Uyo, Nigeria and to establishing the diagnostic value of first trimester pregnancies through detecting normal, abnormal and complicated pregnancies.

## Methods

The ultrasonographic records of pregnant women who were referred to Radiology department of University of Uyo teaching hospital (UUTH), Uyo, Nigeria for ultrasound scans were retrospectively reviewed from the departmental ultrasound record books. The ultrasonographic examinations were performed by Radiologists and Radiology Residents in the department. The ultrasound scan machine used was Toshiba TA311 model, manufactured on 5^th^ October, 2012 with 3.5MHz (convex) probe and 5MHz (trans-vaginal) probe. The period under consideration was from 8^th^ January 2013 to 8^th^ February, 2016. Those who had first trimester ultrasonography were sorted out separately and reserved for this study. Their eligibility criteria were first trimester ultrasonography and positive serum beta human choronic gonadotropin (ßhCG ) level above 1500 IU/ml. When multiple results exist for a patient, the last scan result was chosen for this study. When a patient showed a positive ßhCG test result but did not have confirmed viable pregnancy, she was called for a second control ultrasonography within 10 days. Demographic data like age, parity and special maternal gestational history such as hypertension, diabetes, Rh-Rh isoimmunization, sickle cell diseases and asthma were recorded. Ultrasonographic parameters that were sought were gestational sac diameter (GSD), yolk sac, crown rump length (CRL), fetal cardiac activity, multiple pregnancy, viability, intrauterine ex fetus, anembryonic gestation, subchoronic haematoma, ectopic pregnancy, fetal abnormaly, nuchal lucenceny measurement, miscarriages, trophoblastic pregnancies, adnexal mass, uterine leiomyoma and uterine anomalies. The patients will be divided into different groups according to their sonographic diagnosis. Gestational sac diameter (GSD) and embryonic crown rump length (CRL) were compared with the menstrual age. GSD was calculated as the average of three perpendicular (sagital, transverse, and anteroposterior ) diameters with the calipers placed at the inner edges of the trophoblast [7, 8]. CRL was measured as the greatest length of the embryo on sagital section [7, 9]. Yolk sac diameter was calculated as the average of three perpendicular diameters with the calipers placed at the centre of yolk sac wall [7]. Heart rate was calculated as beats per minute by the software of the ultrasound machine after measurement by electronic calipers of the distance between two heart waves on a frozen M-mode image [7]. Non-visualization of heartbeat in an embryo by 6 weeks or crown–rump length of less than 7 mm after the last menstrual period is suspicious for failed pregnancy [2, 10]. Anembryonic pregnancy (formerly called blighted ovum) is a form of failed pregnancy defined as a GS in which the embryo fails to develop [4, 10]. The embryo, should be observed transvaginally when the GS measures 18-20mm, and transabdominally when the GS measures =25, if absent anembryonic pregnancy is considered [4, 8].

Miscarriage is loss of a recognized pregnancy prior to twenty completed weeks [10]. Threatened miscarriage applies to any pregnancy of less than 20 weeks with abnormal bleeding, pain or contractions, with a closed cervix and subsequent risk of miscarriages [11]. It becomes inevitable miscarriage if the cervical os is markedly open, Missed abortion or miscarriage is when an embryo larger than 5mm or fetus dies but the body does not recognize it to expel it and the placenta may still continue to release hormones. Embryonic demise has replaced the term missed abortion [12]. A complete pregnancy loss is characterized by complete passage of the intrauterine tissue. The cervix is closed and the remaining endometrial thickness is typically less that 15 mm by ultrasound [12]. An incomplete pregnancy loss is characterized by partial passage of the products of conception with clinical or ultrasonographic evidence of retained pregnancy tissue [12]. Retained products of conception are products seen in the endometrial cavity of variable echogenecity and volume seen after spontaneous or therapeutic abortion [11]. Subchorionic haemorrhage or perigestational, hemorrhage is defined as bleeding resulting in marginal abruption with separation of the chorion from the endometrial lining [13]. The presence of an adnexal mass or free pelvic fluid with empty uterus represents ectopic pregnancy until proven otherwise [9]. Nuchal translucency is defined as the presence of a thin translucent area lying between the inner surface of the skin and the soft tissue interface overlying the cervical spine [14]. A measurement ≥3 mm is considered as ‘enlarged’ [14]. Exclusion criterion includes incomplete data, acyesis despite positive ßhCG test. Data was statistically analyzed using SPSS Chicago 13.

## Results

**Figure 1:**
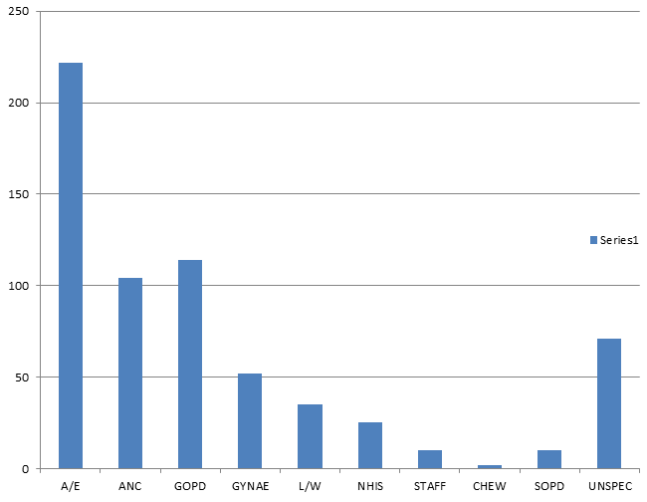
Bar chart showing pattern of referrals to radiology department for first trimester ultasonography

A total of 2438 women had obstetric ultrasonography during the studied period. 26.4% (n-645) of these women had first trimester obstetric ultrasonography. The peak frequency was seen in the 20-29 age range with 52.2% (n-337). This was followed by 30-39 age range with 41.7% (n-269). The least value was in the 50-59 age range with 0.2% (n-1) (Table 1). The commonest first trimester ultrasound findings were viable pregnancy with 42.5% (n-274). This was followed by incomplete miscarriage with 34.3% (n-221). Others in that order were anembryonic gestation 6.5% (n-42) and ectopics 5.9% (n-38). The least common ultrasound findings were trophoblastic pregnancies 0.3% (n-2)(Table 2). The earliest age at which normal viable pregnancy was diagnosed in this study was 5weeks 5days from the last menstrual period. Only 2.19% (n-6) of the normal viable pregnancies were multiple pregnancies and they were all twins. 7.30% (n-20) of normal viable pregnancies had accompanying ovarian corpus luteum cyst of pregnancy. Co-existent maternal uterine leiomyoma(ta) in normal viable pregnancies was seen in 13.87% (n-38) patients. The percentage of abnormal pregnancies was 12.71% (n-82). While the percentage of complicated pregnancies was 44.81% (n-289), the ratio of abnormal and complicated pregnancies to normal viable pregnancies was 1.35:1 (Table 2). The highest number of referrals to Radiology Department for first trimester ultrasonography was from accident and emergency unit with 34.42% (n-222). This was followed by general outpatient department with 17.67% (n-114) and antenatal clinic 16.12% (n-104). The least (n-2, 0.31%) was from children emergency ward (Figure 1).

**Table 1:** Studied population and frequency of first trimester pregnancies in UUTH, Uyo, Nigeria

Age Range	Frequency	Percent
10-19	22	3.4
20-29	337	52.2
30-39	269	41.7
40-49	18	2.8
50-59	1	0.2
Total	645	100.0

**Table 2:** Ultrasonographic Findings of first trimester pregnancies in UUTH

Findings	Frequency	Percent
Ectopics	38	5.9
Threatened Miscarriage	21	3.3
Inevitable abortion	4	0.6
Trophoblastic pregnancies	2	0.3
Incomplete miscarriage	221	34.3
Complete miscarriage	18	2.8
Anembryonic gestation	42	6.5
Embyonic/fetal demise	25	3.9
(Missed abortion)		
Normal pregnancy	274	42.5

## Discussion

The avalanche of positive ultrasonographic findings in this study has more or less typified ultrasound scan as pathway out of first trimester obstetric labyrinth. This positive findings may or may not be desirable to the pregnant mother. It becomes desirable if the positive finding is normal viable pregnancy. Happily, this forms the bulk of our findings constituting 42.5% of our studied population (Table 2). This is lower than 81.3% of single uncomplicated viable pregnancy diagnosis seen in another study [4]. Their higher value may be because of their larger sample size which doubled our studied sample. It may also be due to better patients’ compliance to control second ultrasound scan done 10days after equivocal first scan. The diagnosis of normal viable pregnancy is made on detection of gestational sac (GS), yolk sac and embryo. An intrauterine GS is the first anatomic landmark consistently observed in early pregnancy ultrasonography followed by secondary yolk sac [3, 4, 10].

The earliest age when diagnosis of pregnancy was made in this study was 5weeks 5days. Visualization of GS can be made as early as 4.5 weeks by transvaginal technique [4]. The yolk sac is the first structure able to be visualized within the early gestational sac (chorionic sac) [3]. This yolk sac is a circular structure that measures about 3 to 5 mm in diameter and makes its appearance at about 51/2 weeks of gestation [2, 4]. This is usually by the time the GSD is 8-10mm [3]. Any yolk sac greater than 6 mm is nearly 100% specific for an abnormal pregnancy [3]. The second structure that becomes sonographically visible as echogenic structure within the GS is the embryo seen shortly after the yolk sac [3, 4]. This is at approximately 6 weeks and when the GSD is greater than 16 mm [3]. A definitive pointer to embryonic viability is detection of cardiac activity. Cardiac activity presents as flickering motion and can usually be identified as soon as the embryo is visible which is usually at 6weeks gestational age and when the CRL measures 5mm [2, 11]. By 5 weeks of gestation, the number of gestational sacs within a uterus in multi-fetal pregnancies can be accurately counted and this is called “chorionic sac count” [15, 16]. In this study, 2.19% of viable pregnancies were multiple intrauterine pregnancies with all being twin pregnancies. This gives a ratio 1:45.7 of multiple pregnancy to single pregnancy. This is comparable to the incidence of twin pregnancies in both Jos and Benin of Nigeria. These 2 cities recorded incidence of twin pregnancies of 1:43 [17, 18]. Note that Benin city and our study centre Uyo are in the same geo-politcal zone (South- South) in Nigeria. This incidence in Uyo is lower than the value in a neighbouring city (Calabar) with same aborigine and in same geo-political zone. Calabar has an incidence of 1:37.7 [19].

The incidence in this study is also lower than the incidence in Nnewi (1:29.6) located in neighbouring geo-political zone called South-East [15]. Anembryonic pregnancies, degenerated GS, and intrauterine ex fetus diagnosis are defined as “abnormal pregnancies”[4]. Trophoblastic and ectopic pregnancies are also part of abnormal pregnancies. Viable single pregnancies with fibroids, ovarian cysts, subchorionic hematomas, miscarriages and uterine anomalies are defined as “complicated pregnancies.”[4]. In this study the percentages of abnormal and complicated pregnancies (12.7% and 44.8% respectively) summated together is more than in another study which recorded 18.7% [4]. This is probably due to the fact that their computation was restricted to 9weeks. Detecting abnormal and complicated pregnancies as early as possible can prevent a delay in diagnosis and treatment, thus enhancing both maternal and foetal health[4]. More enlightenment of women of child bearing age is crucial since our data showed low utility of ultrasonography in first trimester. Only 26.4% of women who had obstetric ultrasonography underwent first trimester ultrasonography. And this is cardinal if one remebers that organogenesis, and most malformations are known to arise in this period [4].

Therefore scanning at this period will help to stem down incidence of fetal abnormalities.This non-chalance is also reflected in the pattern of referral for first trimester ultraound scan to our department. 52.1% of first trimester scanned women came from accident/ emergency and general outpatients’ departments (Figure 1). This implies that they were compelled by circumstances to come to hospital like pregnancy complications, pregnancy unrelated ailments or even oblivious of cyesis. Only 16.1% of the women had antenatal booking since they were refered from antenatal clinic. This may imply that early pregnancy booking and subsequent first trimester ultrasonography are poorly embraced by these women. This study revealed two important favourable outcomes. These are viability of pregnancy(commonest findings) (Table 2) and peak reproductive age of second and third decades of life (Table 1). The later is a good tiding as it reduces the frequency of fetal and maternal complications in increased maternal age. These include fetal chromosomal abnormalities (like Down’s syndrome), fetal complications, 5-minute Apgar scores <7, neural tube defect, still birth, perinatal mortality increased neonatal intensive care unit admissions, hypertensive disorders of pregnancy, gestational diabetes, spontaeous abortion, maternal mortality, multiple gestations, subfertility, preterm labour, co-existent maternal leiomyomata, increased incidence of placental abruption, and increased incidence of Caesarian sections [1, 7, 20–27]. The increasing pregnancy rate at advanced maternal age is contemporaneous with the increasing rate of caesarian birth [26]. The significance of this study is that it has shown the need for women to embrace first trimester obstetric ultrasound scan as it will establish viability, abnormal pregnancy and complicated pregnancy. This will definitely guide the management of that pregnancy, mandate apt decision and consequently amelioerate any ensuing burden on the pregnant woman.

This study has some limitations. It is a retrospective study. We believed that the first trimester ultrasound scans were not comprehensively covered by the Sonologists which were mainly junior residents. Certain parameters were either not mentioned or evaded and these include subchoronic haematoma, nuchal translucency and other markers. Non-recording of subchoronic haematoma in the midst of many miscarriges raises a lot of querries. Subchorionic haemorrhage as mentioned previously is bleeding resulting in marginal abruption with separation of the chorion from the endometrial lining. The majority of subchorionic haemorrhages occur in the late first trimester [3]. The paucity of instrumentation with TVS by the Sonologists in this study borne out of patients load and non-consentaneousness of patients must have explained the under-reporting of subchoronic haematoma in this study. TVS is considered the gold standard in the diagnosis and management of subchoronic haemtoma [3]. Consolably, no definite correlation between subchoronic haemorrhage size and pregnancy loss has been confirmed [4]. Similarly, we would have had greater harvest of first trimester ultrasonographies. But for many women who may not have avail themselves of first trimester ultrasound scan on account of scarce resources in their suspected periods of early pregnancy loss.

Those who were advised on control scans after stipulated period probably did not revisit on same account of scarce resources We also believed that uterine and adnexal lesions were under-reported in this study. For example, scanty information was noted on corpus luteum cyst of pregnancy and theca lutein cysts which are known accompaniments of viable pregnancies. Careful investigation of the uterus and adnexae is recommended as part of the routine first trimester evaluation. This is because myomata can grow during pregnancy and obstruct the birth canal while uterine duplication anomalies and septated uterus are associated with a high pregnancy loss rate [4, 9]. Similarly, identification of pregnancy with uterine myomata in the first trimester can avert complications such as first trimester bleeding, anemia during pregnancy, labour dystocia, retained placenta, and the need for neonatal intensive care [4, 28]. Nuchal translucency was never mentioned in all the records of ultrasound findings in this retrospective study. Nevertheless, the above mentioned flaws on the part of these Sonologist will be subsumed under the permissibilty that ultrasonography is operator dependent.

## Conclusion

The commonest first trimester obstetric ultrasonographic finding in Uyo, Nigeria is viable pregnancies and are predominantly single gestation. The peak reproductive ages among women in Uyo, Nigeria are second and third decades and the earliest age of ultrasonographic detection of pregnancy is 5week 5days. Appreciation of utility of first trimester ultrasonography by pregnant women in Uyo is not encouraging as there is low referral for first trimester ultrasonography from antenatal clinics.

**Recommandations:** First trimester obstetric ultrasonography should be free or such expenses defrayed by government or organizations. Departmental template for first trimester obstetric ultrasonography should be drawn as a matter of urgency.

### What is known about this topic

- The diagnosis of normal viable pregnancy is made on detection of gestational sac (GS), yolk sac and embryo;
- Visualization of GS can be made as early as 4.5 weeks by transvaginal technique.

### What this study adds

- The commonest first trimester ultrasound findings was viable pregnancy followed by incomplete miscarriage;
- The earliest age at which normal viable pregnancy was diagnosed in this study was 5 weeks 5 days from the last menstrual period.
